# Gestational choriocarcinoma FIGO stage III, score 8 (high-risk) in 38-year-old woman four weeks postpartum

**DOI:** 10.1515/crpm-2024-0041

**Published:** 2025-06-12

**Authors:** Clara Illi, Wolfgang Henrich, Larry Hinkson

**Affiliations:** Department of Obstetrics, 14903 Charité University Hospital, Berlin, Germany

**Keywords:** gestational choriocarcinoma, gestational trophoblastic neoplasia, retained placental tissue, postpartum bleeding

## Abstract

**Objectives:**

Choriocarcinoma, an aggressive form of gestational trophoblastic neoplasia, can be divided into gestational and non-gestational types, each with distinct biological activity and prognosis. We report a case of gestational choriocarcinoma.

**Case presentation:**

A 38-year-old woman (2 Gravida, 2 Para) presented at our clinic four weeks after her second cesarean section with persistent vaginal bleeding and decreasing hemoglobin to 6.8 mg/L. In the ultrasound examination, retained placental tissue was suspected. Since conservative management with misoprostol was not effective, a curettage was performed. The histopathological result revealed a gestational choriocarcinoma invading the myometrium (CK18 positive, HPL positive, beta-hCG positive, p63 negative, PLAP positive, Ki67 (MIB-1)>80 %). Beta-hCG was 50,607 IU/L at the time of diagnosis. The computed tomography (CT) scan revealed bilateral pulmonary metastases. There were no metastases to the liver, lymph nodes, skeleton or brain. In accordance with FIGO recommendations [stage III, Score 8 (high risk) choriocarcinoma] a multi-drug chemotherapy after EMACO-regimen was started 9.5 weeks postpartum during 14 days for seven cycles. The patient has been in tumor remission since then.

**Conclusions:**

Choriocarcinoma is a differential diagnosis of vaginal peripartum bleeding and might likely be underdiagnosed. Risk factors like a prior hydatidiform mole or abortion, Asian, Indian American, African American or Hispanic ethnicity, advanced maternal age (>40 years), blood group A, or high/increasing beta-hCG postpartum should be taken into consideration.

## Introduction

Gestational trophoblastic disease comprises a spectrum of pre-malignant (complete and partial hydatidiform moles) to malignant conditions (invasive mole, choriocarcinoma, rare placental site trophoblastic tumor, epithelioid trophoblastic tumor). The malignant forms are known as gestational trophoblastic tumors or neoplasias [1].

Choriocarcinoma is a rare and malignant gestational trophoblastic neoplasia with direct invasion into the myometrium and frequent hematogenous metastasis into the lungs, brain, liver, pelvis and vagina, kidney, intestines and spleen. Choriocarcinoma is categorized into two different forms: gestational and non-gestational. Gestational choriocarcinoma is the most common type and might occur after any pregnancy. In 50 % of cases it stems from hydatidiform moles, in 25 % it occurs after abortion or tubal pregnancy, in 25 % it occurs after a normal pregnancy. Only 2–3 % of hydatidiform moles progress to choriocarcinoma [2].

Intraplacental choriocarcinoma as a variant of gestational choriocarcinoma involving both infant and mother is extremely rare. Infantile choriocarcinoma usually becomes symptomatic at the age of 0–6 months with anemia, hepatomegaly, hemorrhagic syndromes. Typical metastases are located in the liver, lungs, brain and skin [3].

In Europe and North America, the incidence of gestational choriocarcinoma is 1:40,000 pregnancies. In Southeast Asia the incidence is higher (3.3–9.2:40,000), as well as in Indian Americans, African Americans and Hispanics [2], 4]. Ethnicity, advanced maternal age, prior spontaneous abortions and molar pregnancies, long-term contraceptive use and blood group A might be risk factors for choriocarcinoma. Non-gestational choriocarcinoma arises from pluripotent germ cells and can appear in women and men, for example in the gonads [5]. It has a much worse prognosis and is also less chemosensitive [5]. Gestational choriocarcinoma usually has a paternal chromosomal pattern while non-gestational choriocarcinoma has the patient’s chromosomal pattern, occasionally with karyotype variations [6].

This case report highlights the differential diagnoses of postpartum hemorrhage and underscores the importance of thoroughly examining the placenta. When a sonographic diagnosis suggests retained placental tissue, it should be followed by regular ultrasound monitoring and beta-hCG surveillance. If bleeding persists or sonographic findings raise concern, surgical removal and histological analysis are necessary to rule out gestational trophoblastic neoplasia.

## Methods

### Staging

Division in low-risk (score<7) and high-risk (score≥7) disease [7]. The prognostic score helps predict the likelihood of developing resistance to single-agent chemotherapy with methotrexate or actinomycin D. A score<7 indicates a low risk of resistance, while a score of ≥7 suggests a high risk. In cases with a high score, the disease is unlikely to be cured with single-agent chemotherapy and requires a multi-agent treatment approach [1], 8].

### Diagnosis and therapy

Treatment is based on classification into risk groups defined by the score and stage.

A low-risk (score<7) and stage I to III choriocarcinoma (FIGO staging Table 1, scoring Table 2) can be treated with a single-agent chemotherapy (methotrexate or actinomycin D). In stage I in patients with concluded family planning, hysterectomy can be considered, but might not obviate the need for chemotherapy.

**Table 1:** FIGO anatomical staging for gestational trophoblastic neoplasia [7].

FIGO classification	Tumor spreading
I	Disease confined to the uterus
II	Disease extending beyond the uterus to genital structures
III	Disease extending to the lungs, with or without known genital tract involvement
IV	Disease invading other metastatic sites

**Table 2:** FIGO 2000 prognostic scoring system for gestational trophoblastic neoplasia.

Risk factor	Score			
	0	1	2	4
Age, years	<40	≥40		
Antecedent pregnancy	Mole	Abortion	Term	
Pregnancy event to treatment interval, months	<4	4 to <7	7 to <13	≥13
Pretreatment hCG, IU/L	<10^3^	10^3^ to <10^4^	10^4^ to <10^5^	≥10^5^
Largest tumor mass, cm	<3	3 to <5	≥5	
Site of metastases	Lung	Spleen, kidney	GI tract	Brain, liver
Number of metastases		1 to 4	5 to 8	>8
Previous failed chemotherapy			Single-drug	>2 drugs

Choriocarcinoma, even in its early stages, can present a higher risk depending on certain risk factors such as an older age, high hCG levels, large tumor size and previous chemotherapy resistance. When stage I choriocarcinoma is classified as high-risk, treatment protocols are typically more aggressive and include multi-agent chemotherapy [1].

A high-risk (score≥7) and stage II to IV choriocarcinoma (FIGO staging Table 1, scoring Table 2) is treated with multi-agent chemotherapy (EMACO-regimen: etoposide, methotrexate, actinomycin D, folinic acid, vincristine, cyclophosphamide), eventually with added adjuvant radiation and surgery.

Since gestational trophoblastic neoplasia are very sensitive to chemotherapy, personalized immunotherapy doesn’t play an important role in the first-line therapy regimen yet. Beta-hCG as a highly sensitive biomarker enables early recognition of therapy resistance and tumor relapse. Vascular targeting agents as well as anti-hCG targeted drugs might be of interest in second-/third-line therapy [1].

### Post-treatment surveillance strategies

After termination of chemotherapy and beta-hCG normalization, beta-hGC monitoring is required and contraception is recommended for at least 12 months. Monitoring should be continued for life [1], 8].

Multi-agent chemotherapy with EMACO might advance the menopause by three years, but fertility is not otherwise affected with 83 % of women becoming pregnant after single-agent or multi-agent chemotherapy. Moreover, the incidence of congenital malformations is not increased. Once a patient becomes pregnant, it is essential to confirm the pregnancy is normal through ultrasound and other appropriate methods. Postpartum, beta-hCG levels should be rechecked at 6 and 10 weeks after delivery to ensure there is no recurrence or new disease [9].

### Prognosis

Gestational and non-gestational choriocarcinomas differ in their prognosis and genotype [6]. The latter has a much worse prognosis and is also less chemosensitive [5].

Patients with a low-risk gestational choriocarcinoma treated with chemotherapy show an almost 100 % survival rate. Patients with a high-risk gestational choriocarcinoma treated with multi-agent chemotherapy have 91–93 % survival rates underlining the fact that stage IV classification or a FIGO scoring>12 are influencing the prognosis significantly [8].

Choriocarcinomas in men with mixed germ cell tumors or as pure carcinomas often have a poor prognosis, particularly in combination with high beta-hCG (>50,000 IU/L) [10].

Intraplacental choriocarcinoma with metastasis to the infant has a very poor prognosis, with <20 % survival [11]. Given the severity of the condition, robust screening protocols and an interdisciplinary approach are essential to identify at-risk pregnancies and infants early, ensuring timely treatment. Neonatal screening should be considered, particularly if there is a history of abnormal placental findings or maternal hCG persistence. Testing the newborn’s hCG levels may provide useful information, as elevated hCG in the infant could indicate metastatic spread of the choriocarcinoma. This can guide further diagnostics and treatment.

## Case presentation

**Figure 1:**
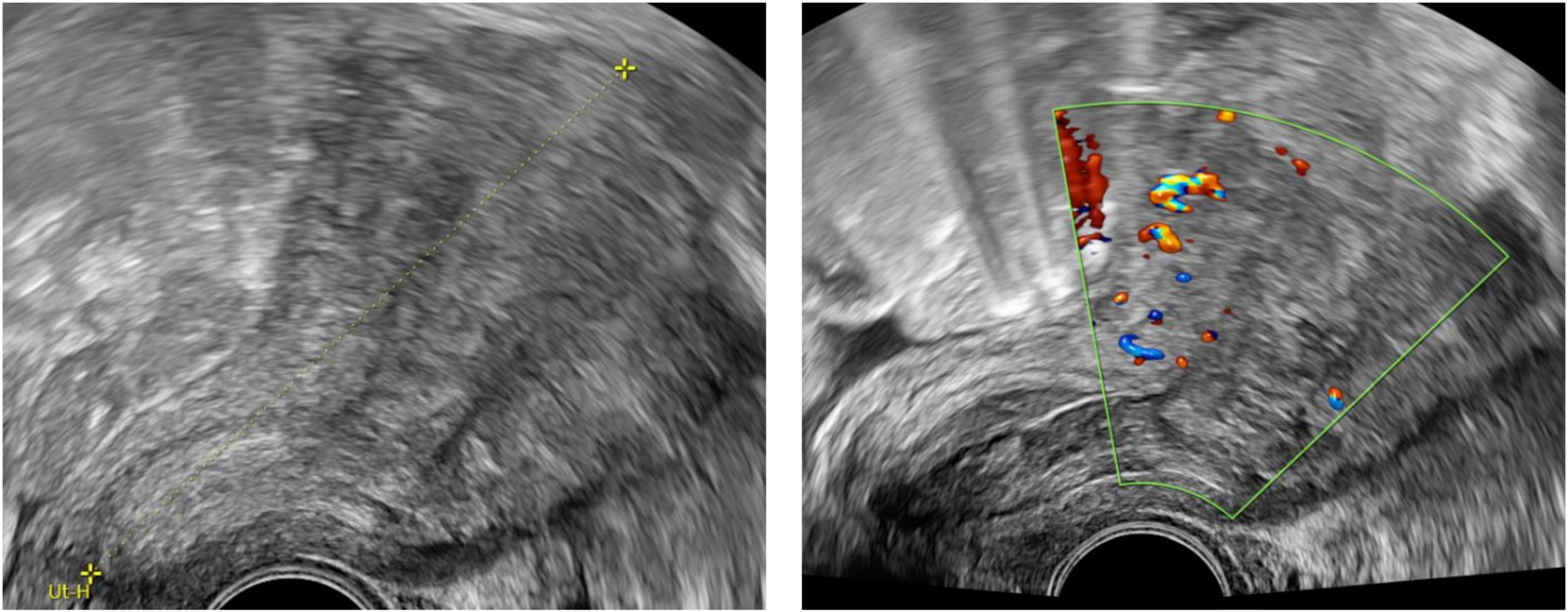
Ultrasound findings four weeks after delivery. The ultrasound findings four weeks after delivery revealed a normal sized uterus with a small 1 × 2 × 3 cm intrauterine diffusely perfused inhomogenous structure.

A 38-year-old woman (2 Gravida, 2 Para) of South American origin presented at our clinic four weeks after her second cesarean section with persistent vaginal bleeding and decreasing hemoglobin from 10.7 to 6.8 mg/L within four weeks. Past medical history included a cesarean section in 2015 due to fetal breech position, tonsillectomy and nasal septum surgery. The patient was breast feeding without complications, had no somatic symptoms and vital parameters were normal. Ultrasound findings included a small 1 × 2 × 3 cm diffusely perfused inhomogenous intrauterine structure (Figure 1) initially assessed to be either a blood clot or retained placental tissue as well as a small amount of approximately 8 mL intraabdominal liquid.

**Figure 2:**
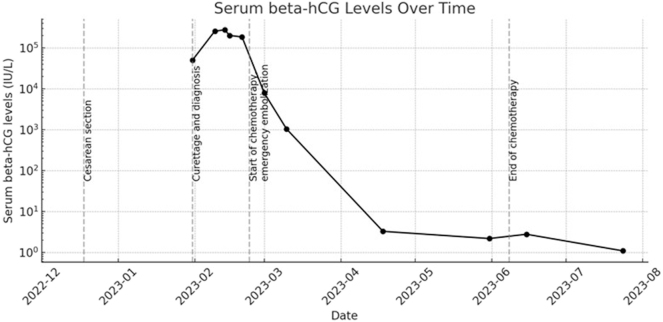
Beta-hCG levels over time. Monitoring of beta-hCG levels with marked medical events of cesarean section, curettage and diagnosis, start and end of chemotherapy, as well as emergency embolization. After the start of chemotherapy beta-hCG quickly decreased back to normal values.

Aside from normocytic normochromic anemia and slightly elevated CRP, routine blood tests were unremarkable. Due to rare anemic symptoms a conservative management with misoprostol and intravenous iron infusions was performed for four days. Finally, due to consistent vaginal bleeding and falling hemoglobin a curettage was performed. The histopathological result of the evacuated tissue revealed a gestational choriocarcinoma invading the myometrium (CK18 positive, HPL positive, beta-hCG positive, p63 negative, PLAP positive, Ki67 (MIB-1)>80 %). Beta-hCG was 50,607 IU/L first measured after the histopathological diagnosis was established (course Figure 2). Unfortunately, beta-hCG values had not been assessed at the first presentation of the patient with postpartum vaginal bleeding. Beta-hCG increased to 276,424 IU/L until chemotherapy was started and quickly went back to normal rates.

**Figure 3:**
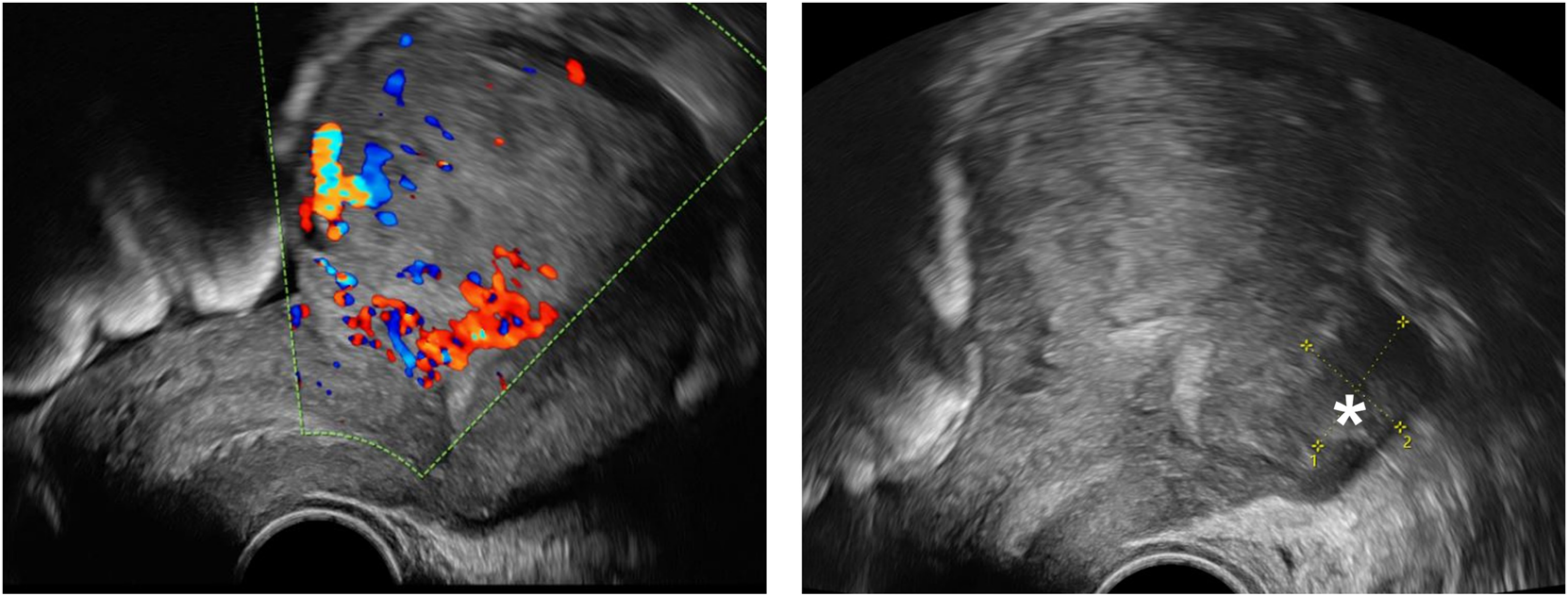
Ultrasound findings after histopathological diagnosis 7.5 weeks after delivery. The ultrasound findings 7.5 weeks after delivery revealed an intrauterine inhomogenous hyperechogenous structure about 4 × 4 × 3 cm in size, now with hyperperfusion and potential invasion of the myometrium (*).

Another ultrasound control 7.5 weeks after delivery revealed an enlarged intrauterine inhomogenous hyperechogenous structure about 4 × 4 × 3 cm in size, now with hyperperfusion and potential invasion of the myometrium (Figure 3).

**Figure 4:**
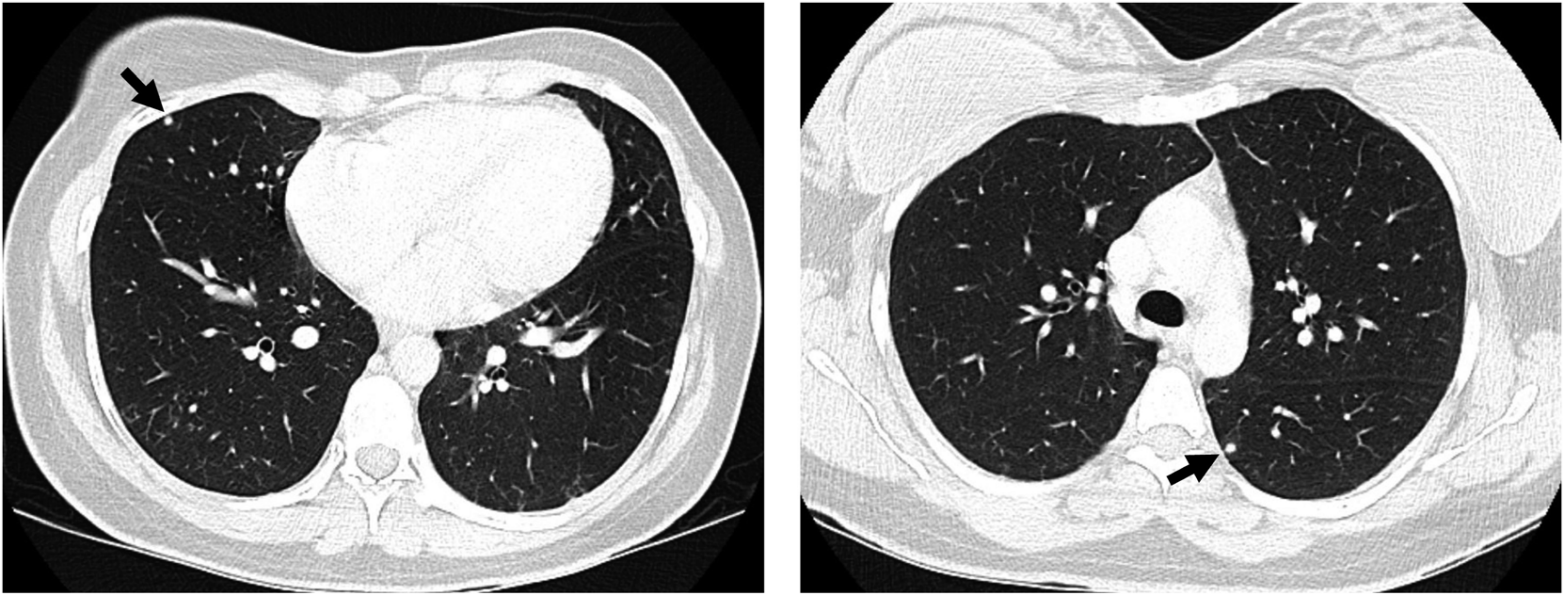
Tumor staging. Computed tomography (CT) scan revealed several small bilateral pulmonary metastases (arrows).

The computed tomography (CT) scan for staging revealed several small bilateral pulmonary metastases. There were no metastases to the liver, lymph nodes, skeleton or brain (Figure 4).

In our case the gestational choriocarcinoma was classified as high-risk (score 8) and FIGO III due to its expansion to the lungs. In the FIGO scoring consists of two points for antecedent pregnancy at term, two points for pretreatment hCG level of 276,424 (10^4^–10^5^) IU/L, two points for a maximal tumor size of >5 cm, and two points for >5 pulmonary metastases, resulting in score of 8. Since the prognostic scoring resulted in a high risk of developing a resistance to single-agent chemotherapy, a multi-agent chemotherapy was chosen.

**Figure 5:**
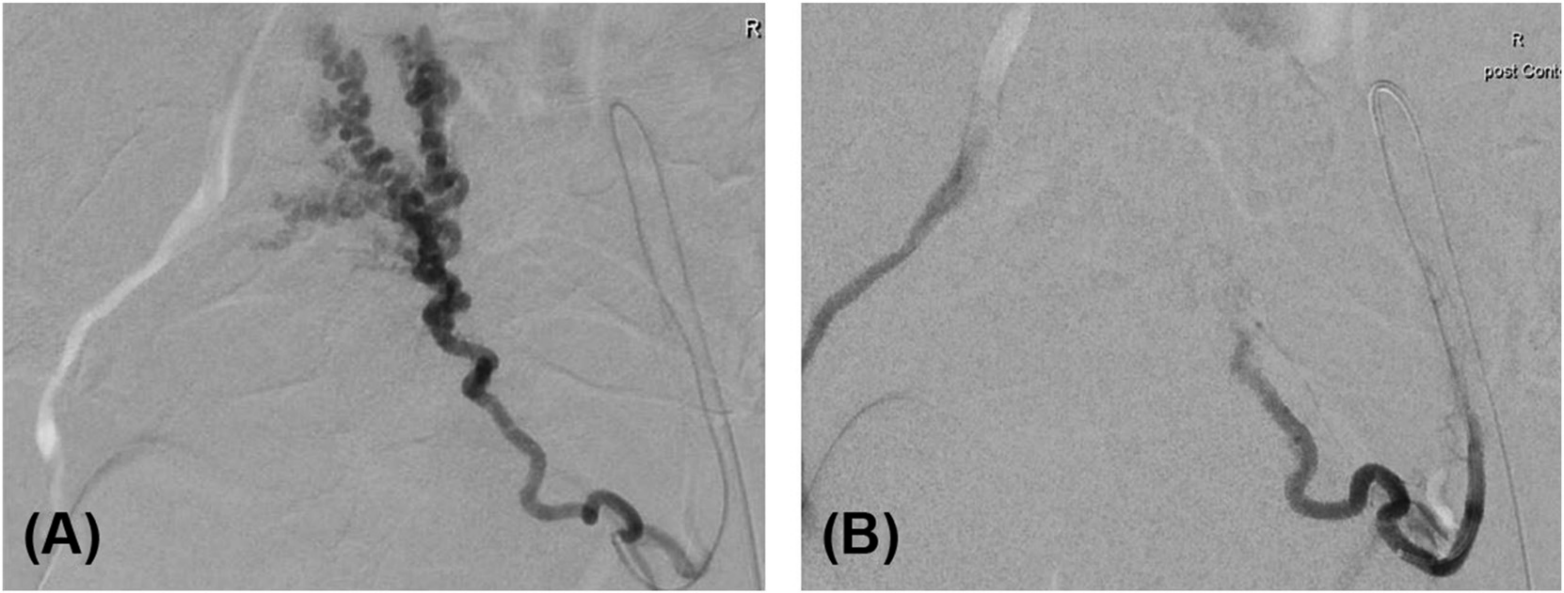
Therapeutic radiological intervention. Emergency transcatheter arterial embolization of the left *A. uterina* was successfully performed due to massive tumor bleeding (A) before intervention and (B) after embolization.

In the course of the inpatient stay a hemodynamically relevant uncontrollable genital bleeding occurred. Therefore, an emergency transcatheter arterial embolization of the uterine artery on both sides was performed (Figure 5). A multi-drug chemotherapy after EMACO-schema was started 9.5 weeks postpartum during 14 days for seven cycles. A CT scan during chemotherapy showed a regression of the tumor mass in the uterus and a complete regression of the pulmonary metastases. A thrombus in the right atrium was identified after the end of chemotherapy, most likely associated with the port catheter. Therefore, anticoagulation with new oral anticoagulants (NOAK) was started. Check-ups since then revealed normal beta-hCG and alpha-fetoprotein levels as well as normal CT scans of the head, thorax, abdomen and pelvis without the suspicious of tumor relapse or metastases. Although not screened, the child did not show any signs of choriocarcinoma.

## Discussion

Choriocarcinoma is a very rare condition, but still an important and life-threatening diagnosis and might likely be underdiagnosed. Ultrasound plays a key role in the diagnosis, but might not be conclusive. Typical findings of thromboplastic neoplasia include an inhomogeneous invasive structure in the uterine cavity with hyperperfusion and multiple cysts. hCG is a tumor-marker and should be measured when placental histology is absent and vaginal bleeding remains persistent. In gestational trophoblastic disease hCG level is usually above those in normal pregnancies and correlates with the progression of the disease. Risk factors like a prior hydatidiform mole or abortion, Asian, Indian American, African American or Hispanic ethnicity, advanced maternal age (>40 years) or blood group A, and measurement of beta-hCG should be taken into consideration. Histopathological diagnosis can only be performed after curettage or biopsy of the metastatic lesions. Although potential etiological factors have been identified, a definitive cause has yet to be determined. It is likely that both exposure to risk factors and intrinsic defects in gametogenesis and fertilization play a role. Classifying types of gestational trophoblastic disease into specific histologic subsets will help to further characterize potential risk factors. To effectively study environmental and genetic factors across different continents and cultures, it is essential to use accurate reproductive denominators, a standardized classification system, and consistent technology to define index cases. The malignant potential of cases identified through these newer technologies also warrants further investigation; until then, ongoing follow-up of these cases remains advisable [9], 12].

Secondary or late postpartum hemorrhage is defined as severe vaginal bleeding between 24 h and 6 weeks after delivery and affects 1–2 % of all pregnancies [13], 14].

Differential diagnoses include uterine atony, infections, retained placental tissue, arteriovenous malformations or a pseudoaneurysma of the uterine artery, primary or metastatic tumors from other organ systems, or subsequent pregnancy shortly after the last [2], 15], 16].

Uterine atony is the most common cause of primary postpartum hemorrhage (80 % of cases), however it is rarely the cause of secondary postpartum hemorrhage.

Infections, such as endomyometritis, are the most common causes of secondary postpartum hemorrhage (67.5 %) [15]. These conditions are associated with fever, uterine tenderness and pain. Management includes antibiotic therapy and supportive care.

Retained placental tissue causes 21.1 % of cases of secondary postpartum hemorrhage [15]. Management includes conservative treatment with uterotonics, if the bleeding is acceptable and beta-hCG levels are normal, or the removal of the retained placental tissue, sometimes requiring surgical procedure such as curettage.

Arteriovenous malformations and pseudoaneurysmas of the uterine artery may require interventional radiological techniques [16]. Risk factors are previous uterine surgery, such as curettage or cesarean section. Surgical interventions, such as a curettage, might increase the bleeding and should be evaluated carefully.

Bleeding from uterine perforation or metastatic lesions may cause abdominal pain, hemoptysis, melena, or signs of intracerebral or lung metastases [2].

Transcatheter arterial embolization in the clinical management of massive genital bleeding resulting from malignant gynecological neoplasms has significant merits before undergoing subsequent tumor therapy and has been described in the literature [17].

In conclusion, secondary postpartum hemorrhage should be carefully evaluated for potential causes, which require different management strategies.

This case report emphasizes the differential diagnoses of vaginal peripartum bleeding and illustrates the importance of a detailed examination of the placenta. The sonographic diagnosis of presumed retained placental tissue should be followed up with regular ultrasound screenings and beta-hCG surveillance. If the bleeding stays persistent or the sonographic findings are suspect, management requires surgical removal and histology to rule out gestational trophoblastic neoplasia.

## What’s already known about this topic?

– Choriocarcinoma is a very rare and aggressive malignant form of gestational trophoblastic neoplasia.
– Choriocarcinoma differs in two different subtypes: gestational and non-gestational. Gestational choriocarcinoma is the most common type and might occur after any pregnancy.

## What does this study add?

– We report the case of a gestational choriocarcinoma in the early postpartum period four weeks after delivery.
– This information can help healthcare providers with the management of postpartum vaginal bleeding and suspected placental residues.
